# Rising prevalence and drug resistance of *Corynebacterium striatum* in lower respiratory tract infections

**DOI:** 10.3389/fcimb.2024.1526312

**Published:** 2025-01-07

**Authors:** Wei Li, Mingyue Gao, Jinyan Yu

**Affiliations:** ^1^ Department of Respiratory and Critical Care Medicine, The Second Hospital of Jilin University, Changchun, Jilin, China; ^2^ Department of Nuclear Medicine, The Second Hospital of Jilin University, Changchun, Jilin, China

**Keywords:** *Corynebacterium striatum*, drug resistance, lower respiratory tract infections, vancomycin therapy, multidrug resistance mechanisms, bacteriophage therapy

## Abstract

*Corynebacterium striatum* (*C. striatum*) is a Gram-positive bacterium commonly colonizing the skin and mucosa in healthy individuals and hospitalized patients. Traditionally regarded as a contaminant, *C. striatum* is now increasingly recognized as a potential cause of clinical infections, especially after the coronavirus disease pandemic. It has emerged as a pathogen implicated in severe infections, including pneumonia, bacteremia, meningitis, artificial joint infections, abdominal infections, and endocarditis. *C. striatum* has been reported in lower respiratory tract infections, mostly as a conditioned pathogen in immunocompromised individuals, particularly in those with chronic structural lung diseases, such as chronic obstructive pulmonary disease, leading to severe pneumonia or exacerbation of the existing disease and high mortality. Additionally, *C striatum* has been implicated in the community-acquired pneumonia among immunocompetent individuals and nosocomial lung infections, with evidence of person-to-person transmission through caregivers. *C. striatum* may exhibit multidrug resistance. Vancomycin, alone or in combination, is currently considered the most effective treatment for *C. striatum*. This review highlights the epidemiological characteristics, drug resistance mechanisms, diagnostics approaches, and treatment options for *C. striatum* lower respiratory tract infections to enhance clinician awareness and improve patient management strategies.

## 1 Introduction

*Corynebacterium* species are characterized by the presence of arabinose, galactose, and meso-diaminovaleric acid in their cell walls. Some strains may also contain corynomycolic acids and metachromatic granules, which serve as reserves of the high-energy phosphate. *Corynebacterium striatum* (*C*. *striatum*) is a non-diphtheriae corynebacterium that is Gram-positive, non-lipophilic, glucose-fermentative or non-fermentative, aerobic or facultatively anaerobic, non-sporulating, and non-motile bacilli. Under a light microscope, it can appear in various forms, including ball, rod-like, and filamentous shapes, occurring singly, in pairs, and/or palisade (Funke et al., 1997; Silva-Santana et al., 2021). *C*. *striatum* is typically a normal flora of the skin and oropharynx and is rarely pathogenic. However, it can opportunistically cause infections in hospitalized patients with underlying conditions (Martinez-Martinez et al., 1997). However, it can be isolated from wound swabs, respiratory specimens, tissue and bone samples, and blood cultures (Funke et al., 1997). In recent decades, there has been increasing reports of invasive infections caused by *C*. *striatum*, including lower respiratory tract infections, intracranial infections, joint infections, and abdominal infections. While they primarily affect immunocompromised individuals, cases have also been reported in patients with normal immune function, albeit rarely (Martinez-Martinez et al., 1997; Lee et al., 2005; Otsuka et al., 2006). *C*. *striatum* mainly causes severe infections in patients with immunocompromised state, including those with end-stage renal disease, structural lung diseases, and advanced cancers. In addition, surgical or invasive procedures, long hospital stays, advanced age, neoplastic disease, organ transplantation, acquired immune deficiency syndrome (AIDS), diabetes, long-term antibiotic use, and procedures requiring continuous or long-term medical devices, such as catheterization, heart valve implantation, and prosthesis placement, for chronic diseases, long intensive care unit stays and reduced hemoglobin levels have also been identified as risk factors associated with C. striatum infection (Martinez-Martinez et al., 1997; Superti et al., 2009; Chen et al., 2012; Verroken et al., 2014; Carvalho et al., 2018; Silva-Santana et al., 2021). There have been documented cases of *C*. *striatum* transmission between patients, leading to serious nosocomial outbreaks. Typically, the isolation of *C. striatum* from a culture is regarded as contamination unless repeated cultures yield consistent results (Kang et al., 2018). Among reported cases of invasive *C. striatum* infections, the most commonly isolated specimens were blood, followed by bone and joint tissues, sputum, and others. The most commonly employed detection method was biochemical analysis alone. However, 16S rRNA gene sequencing and mass spectrometry (mostly via the MALDI-TOF system) were also used as stand alone methods in approximately 17% and 20% of cases, respectively. Biochemical methods combined with MALDI-TOF, or MALDI-TOF combined with 16S rRNA sequencing were also used as detection methods in some cases. In one case, invasive infection caused by *C. striatum* was confirmed using all three methods. Different detection methods can not only identify *C. striatum* as the dominant bacterium causing invasive infection but also determine its drug resistance and virulence genes, providing valuable insights for treatment strategies. One study reported *C. striatum* as the predominant bacterium, with a detection rate of 11.76% in patients with pulmonary infections in the intensive care unit (Zhang et al., 2023). Osawa R and colleagues reported that among patients with bacteremia caused by *C. striatum*, the 90-day mortality rate reached 34% (Yamamuro et al., 2021). Another study indicated that the overall 30-day mortality rate was 34.7% (Abe et al., 2021). Currently, there is no established standard for the treatment of *C. striatum* infections. Given the invasive nature of the bacterium and its high rates of drug resistance—including cases of multidrug resistance—antibiotic selection should be guided by drug resistance gene detection. Vancomycin can be used as a treatment for multidrug-resistant *C. striatum* infections. However, no consensus exists regarding the duration of treatment, which should be tailored based on the severity of the infection and the affected site (Milosavljevic et al., 2021).

In this review, we conducted a comprehensive search of the MEDLINE (PubMed), EBSCO (Discovery Service), SCOPUS, SCIndex (Serbian Citation Index), and Cochrane Central (Wiley Online Library) databases. The search terms included: “corynebacterium striatum”[Supplementary Concept] OR “corynebacterium striatum”[All Fields] OR “corynebacterium striatum”[All Fields]) AND (((“invasibility”[All Fields] OR “invasible”[All Fields] OR “invasion”[All Fields] OR “invasions”[All Fields] OR “invasive”[All Fields] OR “invasively”[All Fields] OR “invasiveness”[All Fields] OR “invasives”[All Fields] OR “invasivity”[All Fields]) AND (“lower airway infect”[All Fields] OR “lower airway infection”[All Fields] OR “lower airway infective”[All Fields] OR”lung infect”[All Fields] OR “lung infection”[All Fields] OR “lung infective”[All Fields] OR “pneumonia”[All Fields]).We summarize the literature on the clinical characteristics, diagnosis, and treatment of *C. striatum*-associated lower respiratory tract infections.

## 2 Clinical characteristics of respiratory infections caused by *Corynebacterium striatum*

In recent years, the incidence of *C. striatum* lower respiratory tract infections has increased, likely owing to advancements in detection technology. Studies have revealed that, following the coronavirus disease (COVID-19) pandemic, the infection rate of *C. striatum* in the lower respiratory tract has markedly increased (Orosz et al., 2022). Furthermore, *C. striatum*, somtimes presented as a superdominant pathobiontic bacterial genus (defined as a genus comprising more than 50% of nasopharyngeal swab sequences), within the nasopharyngeal microbiota, may contribute to severe secondary infections in affected patients (Qin et al., 2020). Common pathogens detected in cases of aspiration pneumonia are *C. striatum*, *Pseudomonas aeruginosa*, *Klebsiella pneumoniae*, and *Candida albicans* (DiBardino and Wunderink, 2015; Neill and Dean, 2019; Xu et al., 2024). Nosocomial infections and outbreaks caused by *C. striatum* primarily occur in the respiratory tract. Research from various countries has confirmed that mechanical ventilation is a risk factor for nosocomial infections (Campanile et al., 2009; Wong et al., 2010). A recent review (1976–2020) analyzing the clinical epidemiology and microbiology of 218 studies confirmed the emergence of multidrug-resistant and multidrug-sensitive *C. striatum* strains in 254 reported cases worldwide, which can cause hospital - and community-acquired infections (Silva-Santana et al., 2021). Prolonged hospital stays, advanced chronic obstructive pulmonary disease, recent antibiotic use, and invasive diagnostic procedures are the most common risk factors for patients with *C. striatum* pneumonia. A positive correlation has been observed between *C. striatum* infections and decreased lung function in chronic obstructive pulmonary disease (forced expiratory volume [FEV1]%) (Renom et al., 2014; Verroken et al., 2014). *C. striatum* resists infection control measures, as it can adhere to non-living surfaces and form biofilms on various medical devices, such as feeding tubes, endotracheal tubes, and ventilators (Souza et al., 2015; Ramos et al., 2019; Lee et al., 2022). The incidence rates of *C. striatum* lower respiratory tract infections are as follows: hospital-acquired pneumonia accounts for 96.3%, including healthcare-associated pneumonia (14.8%) and ventilator-associated pneumonia (11.1%), while community-acquired pneumonia represents 3.7%. *C. striatum* can cause severe pneumonia, which has a mortality rate of up to 60% and is likely to develop into solid cancers, diabetes, and structural lung diseases such as chronic obstructive onary disease (Lee et al., 2022).

Patients with positive cultures from sputum, airway extracts, or bronchoalveolar lavage fluid are typically referred to infectious disease physicians for consultation to determine whether they are pathogenic bacteria. This evaluation includes repeated culture testing, evaluation of infection symptoms and markers, and identification of pathogenic bacteria based on infection guidelines from the Centers for Disease Control and Prevention (Martinez-Martinez et al., 1997; Yamamuro et al., 2021). Invasive infections caused by *C. striatum*are reported more frequently in men than women, with a median patient age of 72 years in cases of severe pneumonia. Notably, 51.9% of the patients are immunocompromised, especially those with structural lung diseases (Martinez-Martinez et al., 1997; Lee et al., 2022). Common clinical symptoms (**Table 1**) include fever (in 80% of cases), dyspnea, productive cough, and phlegm. Serum leukocyte and procalcitonin levels in peripheral blood are often significantly elevated. Pulmonary computed tomography may reveal various nonspecific changes, including nodules and large solid shadows with cavity formation (Severo et al., 2014). Among reported cases of *C. striatum* invasive infections, 75.3% of patients underwent antimicrobial susceptibility testing, primarily due to critical and progressively worsening conditions (Milosavljevic et al., 2021). Many hospitals also conduct routine antimicrobial susceptibility testing on positive pathogenic cultures to guide appropriate clinical therapy. In addition, according to European Committee for Antimicrobial Susceptibility Testing (EUCAST) guidelines, laboratory antimicrobial susceptibility testing for *C. striatum* requires special Settings, as well as broth microdilution, MIC assessment and AGAR diffusion using equine defibrination blood and beta-NAD (Marino et al., 2022).Among patients with severe pneumonia, a high proportion (67%) experienced septic shock, and the 30-day mortality rate was as high as 40.7%. Further analysis of deceased patients revealed a mean FEV1% of 33%, suggesting that decreased lung function may be associated with poor prognosis (Lee et al., 2022).

**Table 1:** Clinical characteristics of respiratory infections caused by Corynebacterium striatum.

Gender	Male >Female
Average Age	72 years
Signs and Symptoms	fever (in 80% of cases), dyspnea, productive cough, and phlegm
Blood Routine	white cell count elevated
PCT	elevated
Chest CT	nonspecific changes, including nodules and large solid shadows with cavity formation
Hospital-acquired pneumonia	96.3% to all pneumonia
Community-acquired pneumonia	3.7% to all pneumonia
Poor prognosis	FEV1% lower than 33%

## 3 Examination method

*C. striatum* is most frequently identified in cultures from clinical specimens, including blood, pus, urine, and pleural effusion (Bao et al., 2017). *C. striatum* as a cause of infection was most often identified exclusively by biochemical methods (Milosavljevic et al., 2021). However, several reports indicate that *C. striatum* may be misidentified or not identified at all using biochemical methods alone. In such cases, alternative techniques like 16S rRNA sequencing are used to confirm the diagnosis (Iaria et al., 2007; Milosavljevic et al., 2021). Application of the VITEK 2 system (bioMerieux,USA), a biochemical method commonly used in clinical practice to detect *C. striatum*, has shown instances of misidentification, including cases where *C. striatum* was mistaken for other bacteria like *Clostridium striatum*. However, the reliability of detection can be improved by including the pathogen in the VITEK system (VITEK^®^2 ANC ID card) database. It can be used to identify *C. striatum*, especially if no alternative test is available (Lee et al., 2011).With advancements in molecular technology, *C. striatum* can be identified using various methods, including 16SrRNA sequencing, complex infection detection chips, metagenomic next-generation sequencing, high-resolution melting analysis, and conventional microbiological tests. These techniques provide simple, rapid, sensitive, and specific options for detecting *C. striatum*, supporting early diagnosis, epidemiological surveillance, and rapid outbreak response (Xu et al., 2021). Mass spectrometry techniques, such as matrix-assisted laser desorption ionization, combined with time of flight and mass spectrometry (MALDI-TOF MS), are also used for bacterial species identification, providing faster and more practical diagnostic solutions (Gomila et al., 2012; Diez-Aguilar et al., 2013). In a hospital outbreak in Belgium, biochemical methods and 16sRNA sequencing techniques were used to identify *C. striatum*. Additionally, MALDI-TOF MS and combined 16S rRNA sequencing have been successfully used in other cases (Verroken et al., 2014). In one case, invasive *C. striatum* infection was identified using a combination of biochemical, MALDI-TOF, and 16S rRNA sequencing techniques. Several studies have shown that gene sequencing is the most reliable method for identifying *C. striatum (* Gomila et al., 2012; Alibi et al., 2015; Suh et al., 2019). Additionally, MALDI-TOF mass spectrometry offers a cost-effective, simple, and reliable alternative for identifying *C. striatum* (Gomila et al., 2012; Suh et al., 2019).

The development of gene sequencing technology has increased detection sensitivity enabling identification of virulence and resistance genes as well as the dominant cloning of bacteria. The core genome multilocus sequence typing (cgMLST) method, based on whole-genome sequencing, can be used to track transmission routes of *C. striatum* infections in hospital settings, which holds significant clinical and epidemiological value (Trost et al., 2011; Torres Lde et al., 2013; Kang et al., 2023). Whole-genome sequencing has also been applied to characterize multidrug-resistant and non-multidrug-resistant clinical isolates of *C. striatum*, providing insights into molecular epidemiology, global transmission, and virulence mechanisms of pathogens (Nudel et al., 2018; Ramos et al., 2018; Wang et al., 2021).

## 4 Virulence and resistance

### 4.1 Virulence

**Figure 1:**
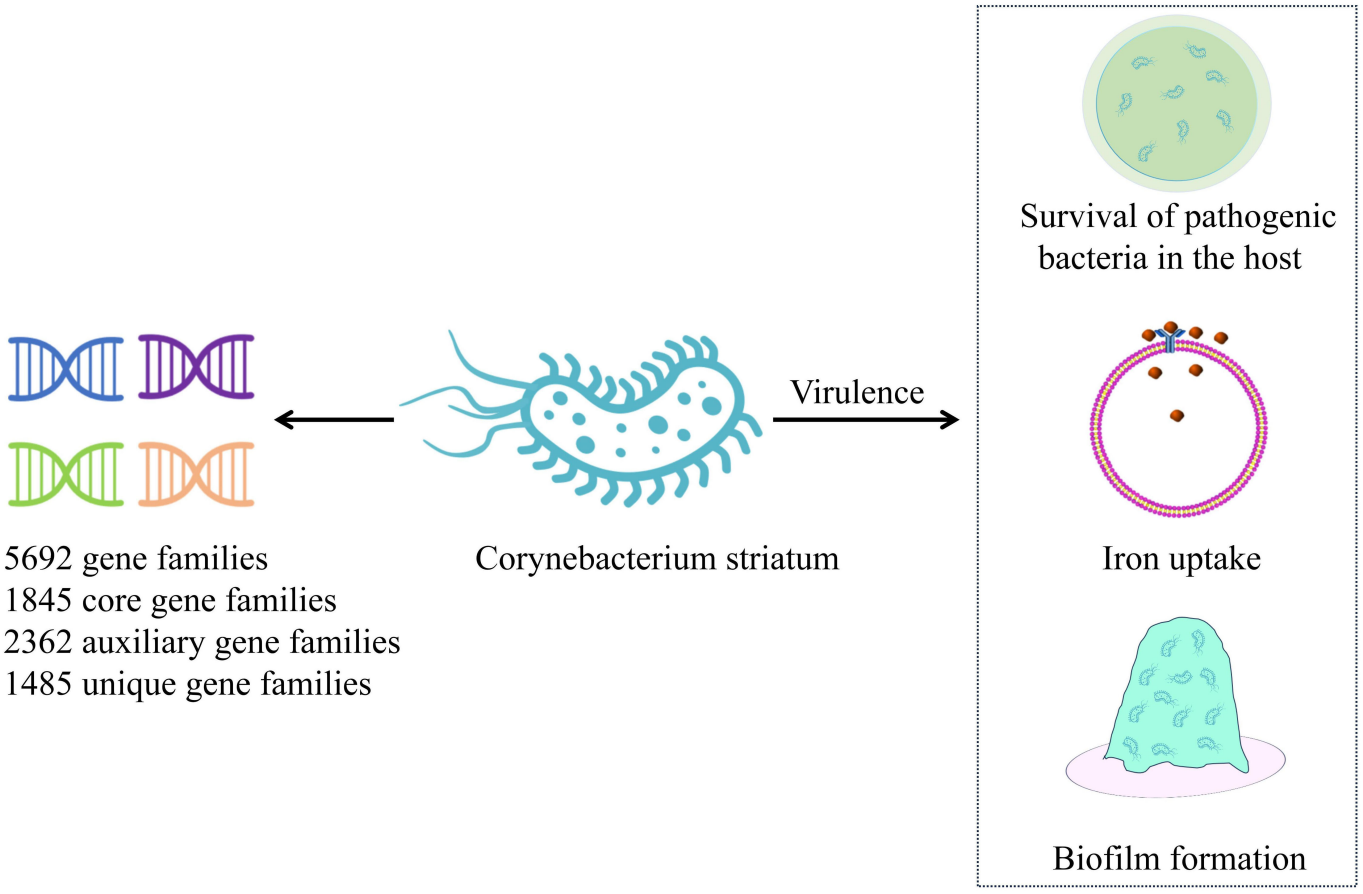
Pan-genome of Corynebacterium striatum. This figure illustrates the pan-genome of recently isolated C. striatum, highlighting key mechanisms of virulence. C. striatum – Corynebacterium striatum.

*C. striatum* is highly invasive and exhibits strong genetic plasticity, with a robust iron acquisition genetic library, independent determinants, and antimicrobial resistance genes (Jesus et al., 2022). *C. striatum* strains from different sources demonstrate high diversity, with those isolated from skin tissues being relatively stable and more conserved. The pan-genome analysis of an emerging multi-drug-resistant *C. striatum* isolate showed an open pan-genome, consisting of 5,692 gene families, 1,845 core gene families, 2,362 auxiliary gene families, and 1,485 unique gene families (**Figure 1**). This analysis further identified 53 resistance genes and 42 virulence factors. Notably, 77.7% of these strains carried two or more resistance genes exhibiting resistance to aminoglycosides, tetracycline, lincomycin, macrolides, and streptomycin. Virulence factors are primarily associated with the survival of pathogenic bacteria in the host, iron uptake, and early biofilm formation (Qiu et al., 2023). The *SpaD* and *SpaE* genes are involved in the formation of pili, which allow the strain to adhere specifically to human pharyngeal epithelial cells (Kang et al., 2014). Additionally, *SrtC* and *SrtB* genes were identified in 48.7% of the strains. *SrtC* is a sorting enzyme associated with pili production through a sorting enzyme mechanism. The *SrtB* gene encodes a collagen-binding protein that binds to the human complement C1q, potentially involved in host immune escape mechanisms and playing an important role in early biofilm formation (Donahue et al., 2014; Chambers et al., 2015). Biofilms have been identified on various human tissues and abiotic surfaces, including medical devices such as catheters, central venous catheters, and endoscopes. The formation of biofilms enhances the survivability of microorganisms under adverse conditions (Ramirez de Arellano et al., 1995; Al Akhrass et al., 2012; Ocalan et al., 2023). Bacteria within biofilms are highly resistant to components of the human immune system and a variety of antibiotics. Moreover, the ability of bacterial cells to transfer genes horizontally is enhanced in biofilm communities, which can promote the spread of antibiotic resistance. The formation of biofilms allows bacteria to adhere to different substrates, enabling their survival in hospital environments. Studies have confirmed that increased biofilm production in C. striatum infection group is a common virulence factor, and it has been observed that C. striatum isolates adhere to various abiotic surfaces to form biofilms *in vitro*, which is the cause of hospital infection outbreak (Souza et al., 2015; Alibi et al., 2021; Ocalan et al., 2023). Research indicates that at 37°C, *C. striatum* is capable of producing biofilms on abiotic surfaces, including polystyrene, glass, and tracheostomy tubes made of polyvinyl chloride, silicone, and stainless steel. These biofilms adhere to both human epithelial cells and abiotic surfaces, enhancing the viability of *C. striatum* in host tissues and the hospital environment. The ability to form biofilms increases the likelihood of hospital-acquired infections while simultaneously elevating bacterial resistance to antimicrobial agents and immune responses (Alibi et al., 2021).Furthermore, several virus-associated proteins, including acyl-CoA carboxylase β-subunits (DtsR1, DtsR2, and AccD3), cell wall-associated hydrolase (CwlH), nonribosomal peptide synthetase (NrpS2), nitric oxide reductase (Nor), resuscitation promoting factors RpfA and RpfB, subtilisin-like serine protease (MycP), SGNh-hydrolase (SgnH), and venom serine protease (Vsp2) identified in *C. striatum* strains are also associated with their pathogenicity (Sangal et al., 2024).

### 4.2 Resistance

**Figure 2:**
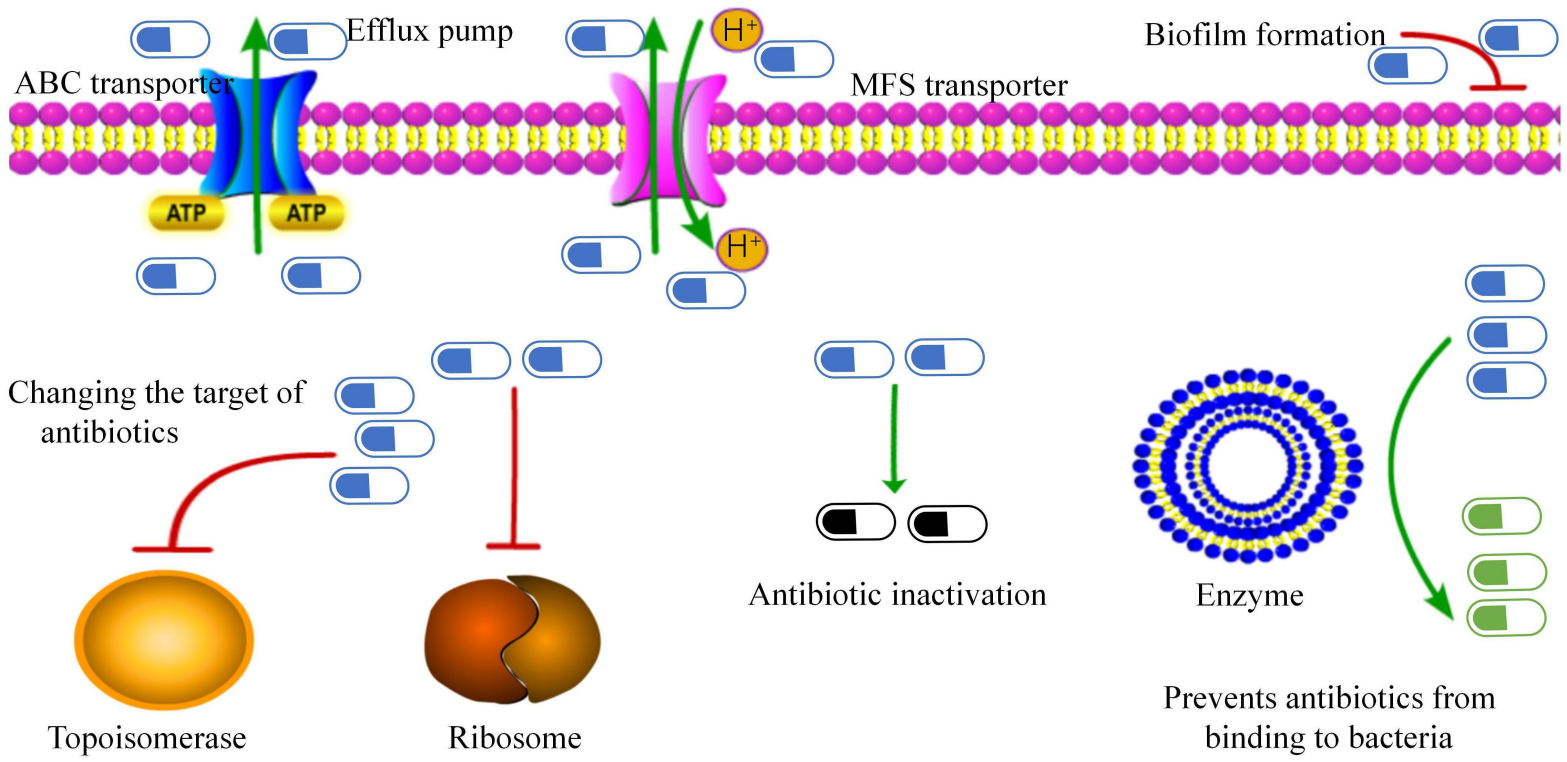
Antibiotic resistance mechanisms in Corynebacterium striatum. This figure presents the identified mechanisms contributing to antibiotic resistance.

Drug-susceptibility testing through bacterial culture remains a widely used classical method for selecting antimicrobial agents against *C. striatum*. Gene sequencing technology can identify drug resistance genes of bacteria, facilitating the understanding of resistance mechanisms in *C. striatum* isolates and the selection of appropriate antimicrobial agents. In the following paragraphs, we discuss the drug resistance mechanisms identified in *C. striatum* isolates reported in this study and the antimicrobial agents to which resistance has been observed.

(A) Efflux pump: The ATP-binding cassette transporter encoded by the *tetA/B* gene transports antibiotics out of the cell membrane, leading to tetracycline and beta-lactam resistance (Kono et al., 1983) (**Figure 2**). Specific major facilitator superfamily transporters and *cmx* genes encoded efflux pumps are associated with chloramphenicol resistance (Leyton et al., 2021).
(B) Biofilm formation: *C. striatum* produces mature biofilms *in vitro*, similar to other pathogenic organisms (Souza et al., 2015). These isolates also exhibited enhanced biofilm formation in the presence of human fibrinogen. Multidrug-resistant strains are strong biofilm producers. Interestingly, isolates from intubated patients showed the highest biofilm production. Other clinical studies on *C. striatum* have also confirmed the production of biofilms (Galimand et al., 2015; Wang et al., 2016; Shariff et al., 2018). The *SrtB* gene encodes a collagen-binding protein in *Clostridium difficile*, which binds to the human complement C1q and may be involved in the host immune escape mechanisms while playing an important role in early biofilm formation (Chambers et al., 2015; Kang et al., 2020; Qiu et al., 2023).
(C) Changing the target of antibiotics: Changes in drug targets due to *gyrA* mutations, including mutations in the quinolone resistance-determining region of the *gyrA* gene (Nudel et al., 2018; Ramos et al., 2018; Asgin and Otlu, 2020), mutations at positions 87 and 91 in the *gyrA* peptide sequence (including those in Ser87Phe, Asp91Ala, and Asp91Gly) (Ramos et al., 2018; Dragomirescu et al., 2020; Ramos et al., 2020). New mutation sites er95Thr, Asp94Ala, Glu88Ala, and Asp87Gly (Qin et al., 2020) convert polar amino acids to nonpolar (except at the 95th position), thereby reducing affinity for fluoroquinolones and preventing their binding to topoisomerase (Nudel et al., 2018). Resistance to macrolides is due to modification of the target by the *erm* gene, which methylates the 23S subunit of the ribosome. Similarly, the *tet* gene, which encodes the ribosome-protective protein tet, confers resistance to tetracycline drugs.
(D) Antibiotic inactivation: The aminoglycoside N-acetyltransferase-coding genes *AAC(6)* and *AAC(6 ‘)-Ib-cr*, which inactivate aminoglycoside antibiotics by acetylating their 6-amino groups, were identified in 57% of the strains (Smith et al., 2017). The *APH(6’)* and *APH(3’)* genes encoding aminoglycoside O-phosphotransferase were detected in 32% of strains, which can inactivate antibiotics, especially streptomycin. The *ANT(3”)* gene family, which encodes a class of aminoglycoside O-nucleotidyl transferases, that are regionally specific according to the 3”-hydroxyl modification of antibiotics, was detected in 38.9% of strains (Qiu et al., 2023). These enzymes inactivate aminoglycoside antibiotics by transferring the AMP group from the ATP substrate to the 3”-hydroxyl group of the compound (Ramirez and Tolmasky, 2010). Beta-lactam resistance is attributed to beta-lactamases encoded by the *bla* and *ampC* genes, which degrade beta-lactam antibiotics (Leyton et al., 2021).
(E) Prevention of antibiotic binding to bacteria: In *Bacillus subtilis*, the loss of phosphoglyceride (PG) in membranes can be fatal; thus, PG is essential for membrane integrity (Hachmann et al., 2011). The *pgsA2* gene of *Fusarium striatum* encodes phosphatidylglycerol synthetase A, responsible for synthesizing diglyceride diphosphate in the PG synthesis pathway (Hines et al., 2017; Goldner et al., 2018; Hagiya et al., 2019). A mutation in the *pgsA2* gene leads to a deficiency of glycerol phosphate in the membrane, preventing daptomycin from binding to the cell membrane. However, Goldner et al. (2018). demonstrated that the striatal C. HLDR phenotype was sufficient in the absence of functional loss of the *pgsA2* gene. This suggests that *C. striatum* is a more persistent and adaptable bacterium than *B. subtilis*. Other resistance mechanisms include various ARGs associated with mobile genetic elements such as plasmids, integrons, insertion sequences, and transposons (Nesvera et al., 1998; Tauch et al., 2003; Hennart et al., 2020). The resistance of *C. striatum* is primarily determined by transposons, insertion sequences, and plasmids. The Macrolide-lincosamide-streptogramin B (MLS) phenotype is a common resistance mechanism in coagulase-negative staphylococci and also in *C. striatum* related to the *erm(X)* gene transported by the transposon Tn5432 (Szemraj et al., 2018; Szemraj et al., 2019).

The resistance in *C. striatum* is dynamic and expansive, which is also related to the transposon Tn5432, the MLS phenotype, and the insertion sequence, among others (Nesvera et al., 1998; Hennart et al., 2020). This leads to cross-infection, spread, and evolution of pathogenic bacteria in hospitals, necessitating that hospital staff be aware of nosocomial infections caused by *C. striatum* (Souza et al., 2020; Wang et al., 2022).

## 5 Treatment

Several studies have documented resistance in isolated strains of *C. striatum*. As mentioned earlier, bacterial culture and drug susceptibility testing can be effective methods for guiding antibiotic selection. Additionally, gene sequencing technology can accurately identify drug-resistance genes in isolated strains and is increasingly being utilized to inform clinical decisions regarding antibiotic selection.

In China, isolates were collected from three hospitals across three regions, with 260 isolates from patients with respiratory infections. Nearly all isolates (96.2%, 250/260) showed multidrug resistance, although they remained sensitive to vancomycin or linezolid, which aligns with findings from other countries (Zhang et al., 2023). Of these strains, 77.7% harbored two or more resistance genes and showed primary resistance to aminoglycosides, tetracycline, lincomycin, macrolides, and streptomycin (Qiu et al., 2023). Another study reported that 54 C*. striatum* isolates exhibited multidrug resistance to three or more antibiotics, with a resistance rate of 85.2% to lincomines and 93.5% to quinolones and tetracyclines. Sensitivity to vancomycin and linezolid was 100% (Wang et al., 2022).

Given its efficacy, vancomycin should be considered the antibiotic of choice for treating *C. striatum* infections. Vancomycin monotherapy, or combined with other antibiotics, such as piperacillin-tazobactam, may be the most prudent approach for multidrug-resistant *C. striatum* lung infections. Based on resistance predictions, vancomycin may remain one of the few effective agents currently in use by 2030 (Tarr et al., 2003; Babay and Kambal, 2004; Orosz et al., 2022). Alternatively, linezolid, teicoplanin, or daptomycin may be considered for treating severe lung infections caused by *C. striatum*, while amoxicillin-clavulanate may be used in mild infection cases (Milosavljevic et al., 2021).

However, a study has reported no difference in hospital mortality among patients with severe pneumonia who received antimicrobials targeting *C. striatum*, including vancomycin and linezolid. This outcome was similar to that in patients who did not receive anti-infective treatment. This may be attributed to the severity of illness in the *C. striatum* infection group, which included more critically ill patients with APACHE II scores >15. A subgroup analysis indicated that vancomycin or linezolid use reduced all-cause mortality (Zhang et al., 2023).

Daptomycin is also effective in resistant Gram-positive bacterial infections; however, reports of daptomycin-resistant *C. striatum* strains have emerged, potentially leading to treatment failure (Chauvelot et al., 2020).

Dalbavancin may serve as a successful and safe alternative for *C. striatum* infections, particularly in soft tissue infections. Approved for bacterial skin and soft tissue infections, dalbavancin’s optimal dosing and interval remain to be determined, and therapeutic drug monitoring may help in guiding treatment (Soderquist et al., 2023; Camara-Rodriguez et al., 2024).

Recently, new treatments for multidrug-resistant *C. striatum* infections have also shown promise. CSP1, a novel temperate bacteriophage and the first phage identified to target *C. striatum* strains could offer new possibilities in bacteriophage therapy research (Wang et al., 2024). Niclosamide, which reduces biofilm viability in a dose-dependent manner, has been approved for degrading biofilm biomass, and drastically reducing cell viability. Therefore, niclosamide is emerging as a promising therapeutic agent against multidrug-resistant *C. striatum* infections (Folliero et al., 2022).

## 6 Conclusion

Reports indicate that *C. striatum* lower respiratory tract infections are increasing, particularly during the COVID-19 pandemic. *C. striatum* can cause both community-acquired and hospital-acquired pneumonia in healthy and immunocompromised individuals. It can lead to severe pneumonia with high mortality, particularly in patients with structural lung diseases, such as chronic obstructive pulmonary disease. The prognosis of *C. striatum* pneumonia correlates with a decline in FEV1%. Despite potential detection errors, biochemical methods remain reliable for clinical identification of *C. striatum*. It has been reported that gene sequencing technology (16sRNA) is the most reliable method for detection of invasive infections caused by this pathogen, and can be considered as the gold standard for diagnosis. Whole-genome sequencing offers additional insights by identifying bacterial virulence factors and drug resistance genes. Furthermore, MALDI-TOF mass spectrometry is increasingly being used in clinical settings due to its rapid, cost-effective, and reliable detection capabilities. Isolated strains of *C. striatum* exhibit high rates of drug resistance, complex resistance mechanisms, including efflux pumps, biofilm formation, target modification of antibiotics, antibiotic inactivation, and prevention of antibiotic binding to bacteria. These factors also contribute to its potential for nosocomial transmission. At present, there is no standardized antibiotic regimen for treating lower respiratory tract infections caused by *C. striatum*. Antibiotic selection should be guided by the severity of the patient’s condition, as well as the virulence and drug resistance profile of the isolated strain. Vancomycin remains the most effective treatment, either alone or in combination with other agents. For mild cases, drugs such as piperacillin and sulbactam may be considered. New therapies, including temperate bacteriophages such as CSP1, hold promise for managing drug-resistant *C. striatum* in the future.
